# Confocal laser endomicroscopy in glial tumors—a histomorphological analysis

**DOI:** 10.1007/s10143-024-02286-3

**Published:** 2024-01-24

**Authors:** Karen Radtke, Walter J. Schulz-Schaeffer, Joachim Oertel

**Affiliations:** 1 Klinik für Neurochirurgie, Medizinische Fakultät, Universität des Saarlandes, /Saar, 66421 Homburg, Germany; 2 Institut für Neuropathologie, Medizinische Fakultät, Universität des Saarlandes, /Saar, 66421 Homburg, Germany

**Keywords:** Confocal laser endomicroscopy, Autofluorescence, Glioma, Neurooncology, Brain tumor

## Abstract

**Objective:**

The extent of resection and neurological outcome are important prognostic markers for overall survival in glioma patients. Confocal laser endomicroscopy is a tool to examine tissue without the need for fixation or staining. This study aims to analyze gliomas in confocal laser endomicroscopy and identify reliable diagnostic criteria for glial matter and glial tumors.

**Material and methods:**

One-hundred-and-five glioma specimens were analyzed using a 670-nm confocal laser endomicroscope and then processed into hematoxylin-eosin-stained frozen sections. All confocal images and frozen sections were evaluated for the following criteria: presence of tumor, cellularity, nuclear pleomorphism, changes of the extracellular glial matrix, microvascular proliferation, necrosis, and mitotic activity. Recurring characteristics were identified. Accuracy, sensitivity, specificity, and positive and negative predictive values were assessed for each feature.

**Results:**

All 125 specimens could be processed and successfully analyzed via confocal laser endomicroscopy. We found diagnostic criteria to identify white and grey matter and analyze cellularity, nuclear pleomorphism, changes in the glial matrix, vascularization, and necrosis in glial tumors. An accuracy of > 90.0 % was reached for grey matter, cellularity, and necrosis, > 80.0 % for white matter and nuclear pleomorphism, and > 70.0 % for microvascular proliferation and changes of the glial matrix. Mitotic activity could not be identified. Astroglial tumors showed significantly less nuclear pleomorphism in confocal laser endomicroscopy than oligodendroglial tumors (*p* < 0.001). Visualization of necrosis aids in the differentiation of low grade gliomas and high grade gliomas  (*p* < 0.002).

**Conclusion:**

Autofluorescence-based confocal laser endomicroscopy proved not only useful in differentiation between tumor and brain tissue but also revealed useful clues to further characterize tissue without processing in a lab. Possible applications include the improvement of extent of resection and the safe harvest of representative tissue for histopathological and molecular genetic diagnostics.

## Introduction

Gliomas are a group of mostly malignant, diffusely infiltrating intracranial neoplasms. In an age where new adjuvant treatment options are being tested to possibly provide personalized treatment, surgical resection is still the first step to confirm diagnosis and reduce tumor volume.

The extent of resection (EOR) has been proven to be crucial for overall and progression-free survival for glioblastoma [1], regardless of other factors such as age, Karnofsky Performance Status (KPS), and chemotherapy [2]. EOR has also been identified as important for overall survival in patients with low-grade gliomas [3, 4]. Another independent prognostic factor for both low-grade and high-grade gliomas is residual tumor volume [4–6]. These studies prove the importance of successful surgical treatment for standardized care.

After surgical resection, histopathological analysis is of utmost importance for the patient and their prognosis. While new criteria, especially genetic analysis, have gained importance in the 2021 WHO Classification, the classical morphological features of glial tissue and glial tumors are still evaluated by neuropathologists under a microscope.

Patients with WHO Grade 1 and 2 gliomas, commonly referred to as low-grade gliomas (LGGs), live much longer than patients with high-grade gliomas (HGGs), WHO Grades 3 and 4. Malignant features of glial tumors include visible mitotic activity, microvascular proliferation, and necrosis. Features such as nuclear pleomorphism and cellularity might help indicate the cell of origin. The diagnosis then determines the patients’ treatment options.

Confocal laser endomicroscopy has emerged in the last decade as a tool to analyze tissue without the need for processing into paraffin or frozen sections [7–9]. Modern confocal laser endomicroscopes have been approved for in vivo use and have been evaluated in their use in differentiating between tumor and non-tumorous tissue in intracranial neoplasms [10, 11]. Studies have shown the possibility to differentiate between glial and mesenchymal tumors as well as metastases [11–14]. Still, explicit descriptions of characteristic features to allow standardized interpretation have not been made yet. Additionally, most studies focus on the behavior of fluorescent dyes instead of standard histological features, which results in stress for the patient and surgical staff. Thus, our aim is to analyze the visualization of histomorphological features of glial matter and gliomas via autofluorescence-based CLE and establish criteria to identify these features at any given time, independent of the examiner and their previous experience with confocal laser endomicroscopy (CLE) or neuropathological glioma diagnostics. Finally, we want to discuss the importance of fluorescent dyes in confocal laser endomicroscopy.

## Material and methods

### Study design

Thirty-six patients were enrolled in this study in a prospective manner to analyze the visualization of glioma tissue and its histomorphology via autofluorescence-based confocal laser endomicroscopy. Enrollment criteria were brain lesions suspicious of glioma in neuroimaging or medical history of prediagnosed glioma scheduled for surgery and age > 18 years.

Each patient consented to both surgery and participation in this study. This study was approved by the responsible Ethics committee (Ethikkommission des Saarlandes, No. 209/2021).

Tissue was examined ex vivo via CLE and as hematoxylin and eosin-stained (H&E) frozen sections.

### Tumor biopsy acquisition

Tissue specimens that were suspicious of glial tumors and were to be removed as part of standard tumor resection were collected for this study. These samples were handled separately from routine diagnostics and were not used to make a final neuropathological diagnosis. A senior neurosurgeon determined how many biopsies were available for CLE examination. If possible, more than one sample of each patient was collected. No macroscopically healthy brain tissue or tissue that would have otherwise remained in situ was resected for the purpose of this study.

### Confocal laser endomicroscope—technical note

For CLE imaging, the confocal laser endomicroscope EndoMag1 by Karl Storz SE & Co. KG (Tuttlingen, Germany) was used. It consists of an endoscope and an adjunct computer with software by Heidelberg Engineering GmbH (Heidelberg, Germany). The endoscope itself is made up of a rigid rod lens system that is not approved for in vivo use. The outer diameter of the probe is 0.5 cm, its length is 32.3 cm. The laser emits red light with a wavelength of 670 nm. The EndoMag1 has a round field of view with a diameter of 300 μm. It is able to obtain 40 images per second and thus provides real-time imaging of analyzed tissue. The maximum depth of penetration of the tissue is 300 μm. The focal plane can be changed manually in a range of 80 μm. According to the manufacturer, lateral resolution is 1–2 μm, axial resolution is 2 μm. The illumination of the field of view can be regulated automatically by the software or manually. The software allows the user to store images for different patients, interrupt and continue examinations, or reexamine patients at any time.

### CLE image acquisition

CLE imaging was performed ex vivo directly after the collection of the tumor samples in the operating theater. The specimens were placed under the probe of the CLE, and the tip of the probe was gently attached to the specimen surface. Excessive blood was carefully flushed away with isotonic saline solution. No topical staining agent was used. Each specimen was then scanned in different depths, then turned around and scanned again. Images of representative areas and remarkable structures were captured and stored.

### Neuropathological processing

Immediately after CLE imaging, each biopsy was embedded in tragacanth (Sigma-Aldrich, St. Louis, MO, USA) and frozen in 2-methylbutane (VWR International, Rosny-sous-Bois cedex, France) pre-cooled in liquid nitrogen. The specimens were then cut into 6 μm frozen sections with a cryostat-microtome (Leica CM1850, Leica Microsystems Ltd., Wetzlar, Germany), left to dry for 1 h, and fixed with 10 % formaldehyde + 60 mmol calcium chloride (Merck KGaA, Darmstadt, Germany). The frozen sections were then stained using hematoxylin-and-eosin (H&E) (Merck KGaA, Darmstadt, Germany) and dehydrated in an ascending alcohol series and xylole.

### Analyzing frozen sections and correlating CLE images

A grading system for each specimen was developed by a senior neuropathologist. We determined nine criteria to be evaluated for each specimen (Table 1) both by microscope and CLE. Each feature was then assessed separately with both modalities. The criteria were then dichotomized and compared for their visualization via microscope and CLE. In the same manner, we analyzed the visualization of the histomorphological criteria in astro- versus oligodendroglial and low-grade versus high-grade gliomas.

**Table 1:** Evaluation criteria for confocal images and frozen sections

Criteria	0		1	
Grey matter	No		Yes	
White matter	No		Yes	
Rarefaction of the glial matrix	No		Yes	
Hypercellularity	No	Slight	Moderate	Extensive
Nuclear pleomorphism	No	Slight	Moderate	Extensive
Hypervascularization	No		Yes	
Mitotic activity	No		Yes	
Necrosis	No		Yes	
Tumor	No		Yes	

## Results

### Patient and tumor sample characteristics

Thirty-six patients were enrolled in this study (Table 2). Tumor samples were harvested in 38 surgical procedures (one patient underwent surgery thrice). Of these 36 patients, 21 were male and 15 female. The mean age was 55.0 years. A total of 125 glioma specimens were harvested, mean number of samples per surgery was 3.3 (range 1–7). Four-thousand-forty-seven confocal images were acquired, mean number per biopsy was 34 (range 4–80).

**Table 2:** Overview of patient characteristics and tumor samples

Sex	Patients (*n* = 36)		
Male	21	58.33 %	
Female	15	41.67 %	
Age	Patients (*n* = 36)		
Mean age in years	55.0		
Intraoperative rapid frozen section analysis	Patients (*n* = 36)		
Glial tumor	37	97.37 %	
Necrosis	1	2.7 %	
Neuropathological diagnosis	Patients (*n* = 38)		Specimens (*n* = 125)
Astrocytic tumor	30	76.32 %	*102*
Pilomyxoid astrozytoma (not graded)	1		3
Subependymal giant cell astrocytoma WHO grade 1	1		2
Ganglioglioma WHO grade 1	1		3
Astrocytoma WHO grade 2	5		20
Astrocytoma WHO grade 3	7		21
Astrocytoma WHO grade 4 (IDH-mutant)	2		8
Glioblastoma WHO grade 4 (IDH-wildtype)	13		45
Oligodendroglial tumor	8	21.05 %	*23*
Oligodendroglioma WHO grade 2	5		14
Oligodendroglioma WHO grade 3	3		9

In 37 out of 38 cases, the standard rapid frozen section analysis made by a senior neuropathologist was glial tumor. In one case, only tumor necrosis was identified.

All included tumors were gliomas. Thirty of 38 tumors were astroglial tumors, including one pilomyxoid astrocytoma, one subependymal giant cell astrocytoma WHO Grade 1, one ganglioglioma WHO Grade 1 with astroglial differentiation of the glial cell portion, 5 astrocytomas WHO Grade 2, 7 astrocytomas WHO Grade 3, 2 IDH1-mutated astrocytomas WHO Grade 4 and 13 IDH-wildtype glioblastomas WHO Grade 4. 8 of 38 tumors were of oligodendroglial origin, 5 WHO Grade 2 oligodendrogliomas and 3 WHO Grade 3 oligodendrogliomas.

### Histomorphological findings in frozen section versus CLE

#### Grey matter

**Fig. 1:**
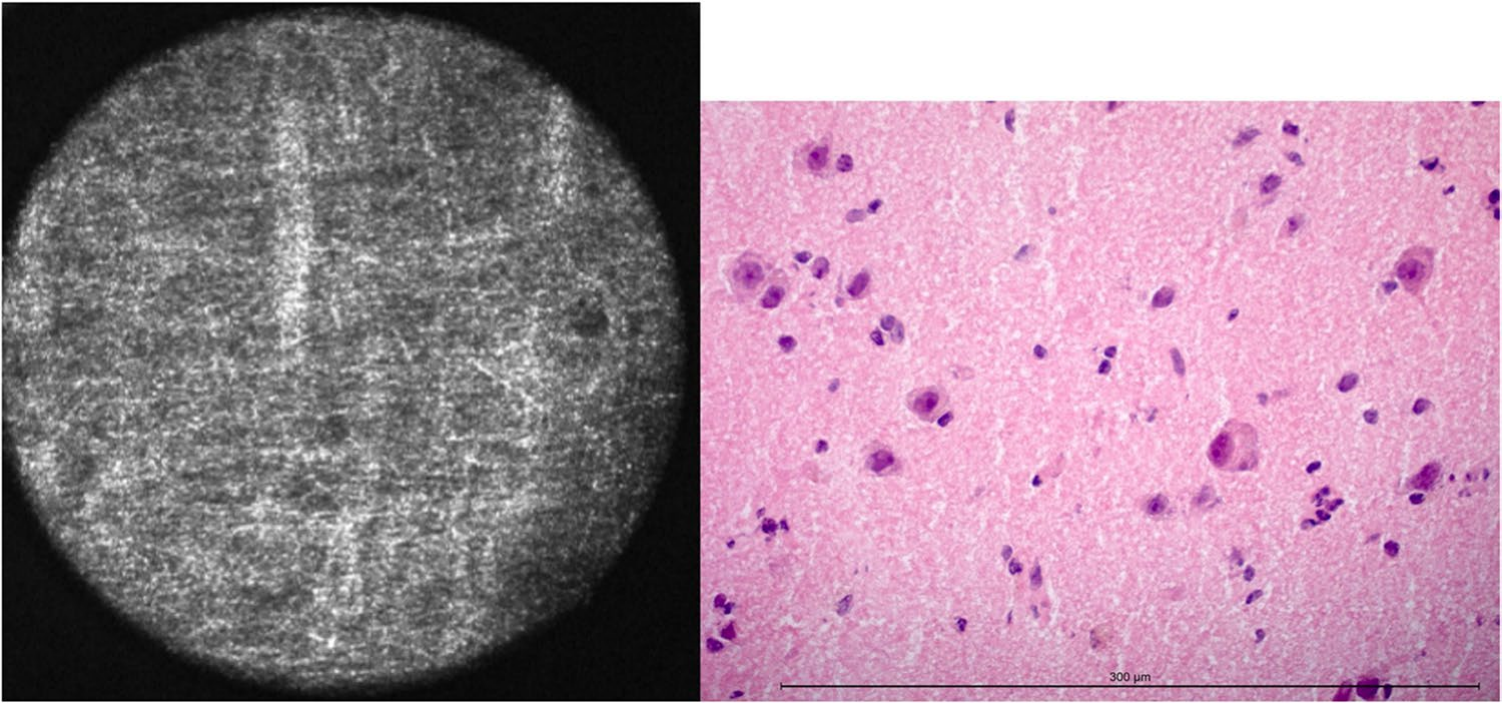
Grey matter in CLE and H&E-frozen section. The diameter of the confocal image is 300 μm. The length of the measuring bar is 300 μm

CLE showed cortical grey matter nearly geometrically organized (Fig. 1), which fits the general understanding of axons drawing from the cortex to the subcortical white matter orthogonally to the brain surface and dendrites interconnecting neurons. The visualization of this geometrical pattern was dependent on the orientation of the CLE-probe to the tissue sample. Round signal losses between 10 and 30 μm were interpreted as cortical neurons. Other cell types such as glial cells or cells of the immune system could not be securely identified via CLE.

Twenty-nine specimens contained grey matter, and 96 did not. Via CLE, 112 of 125 were identified correctly, resulting in an accuracy of 94.4% for grey matter (Table 3).

**Table 3:** Accuracy, sensitivity, specificity, positive, and negative predictive values for each histologic criterion

Feature	Accuracy	Sensitivity	Specificity	PPV	NPV
Grey matter	94.44 %	79.31 %	92.71 %	76.67 %	93.68 %
White matter	88.8 %	84.62 %	91.75 %	73.33 %	93.68 %
Glial matrix changes	73.98 %	90.91 %	64.56 %	58.82 %	92.73 %
Hypercellularity	90.4 %	93.33 %	82.86 %	93.33 %	82.86 %
Nuclear pleomorphism	84.0 %	87.06 %	77.5 %	89.16 %	73.91 %
Hypervascularization	79.2 %	83.61 %	75.0 %	76.12 %	82.76 %
Mitotic activity	–	–	–	–	–
Necrosis	92.0 %	87.5 %	92.66 %	63.64 %	98.06 %

#### White matter

**Fig. 2:**
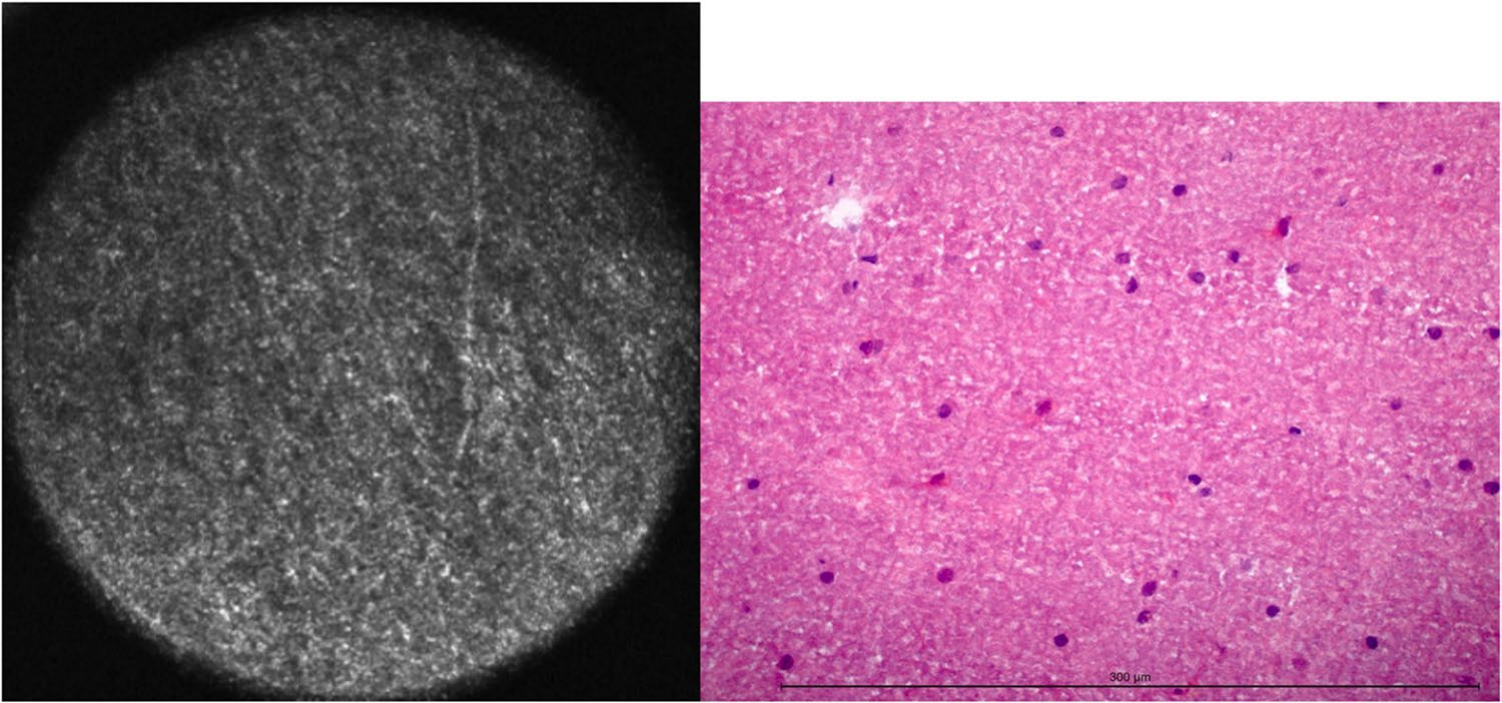
White matter in CLE and H&E-frozen section. The diameter of the confocal image is 300 μm. The length of the measuring bar is 300 μm

In CLE imaging, white matter appeared more homogenously than grey matter and with a more intense signal (Fig. 2). Fibrous structures were less distinctive, in total white matter looked more diffuse. Small signal losses were interpreted to be the equivalent of glial cells due to their predominance in white matter, but not surely identified as either astro- or oligodendroglial cells. Other cell types such as lymphocytes could not be detected via CLE.

Twenty-eight samples contained white matter, and 97 did not. 111 samples were diagnosed correctly via CLE. In total, white matter was detected with an accuracy of 88.8 % (Table 3).

#### Rarefaction of the glial matrix

**Fig. 3:**
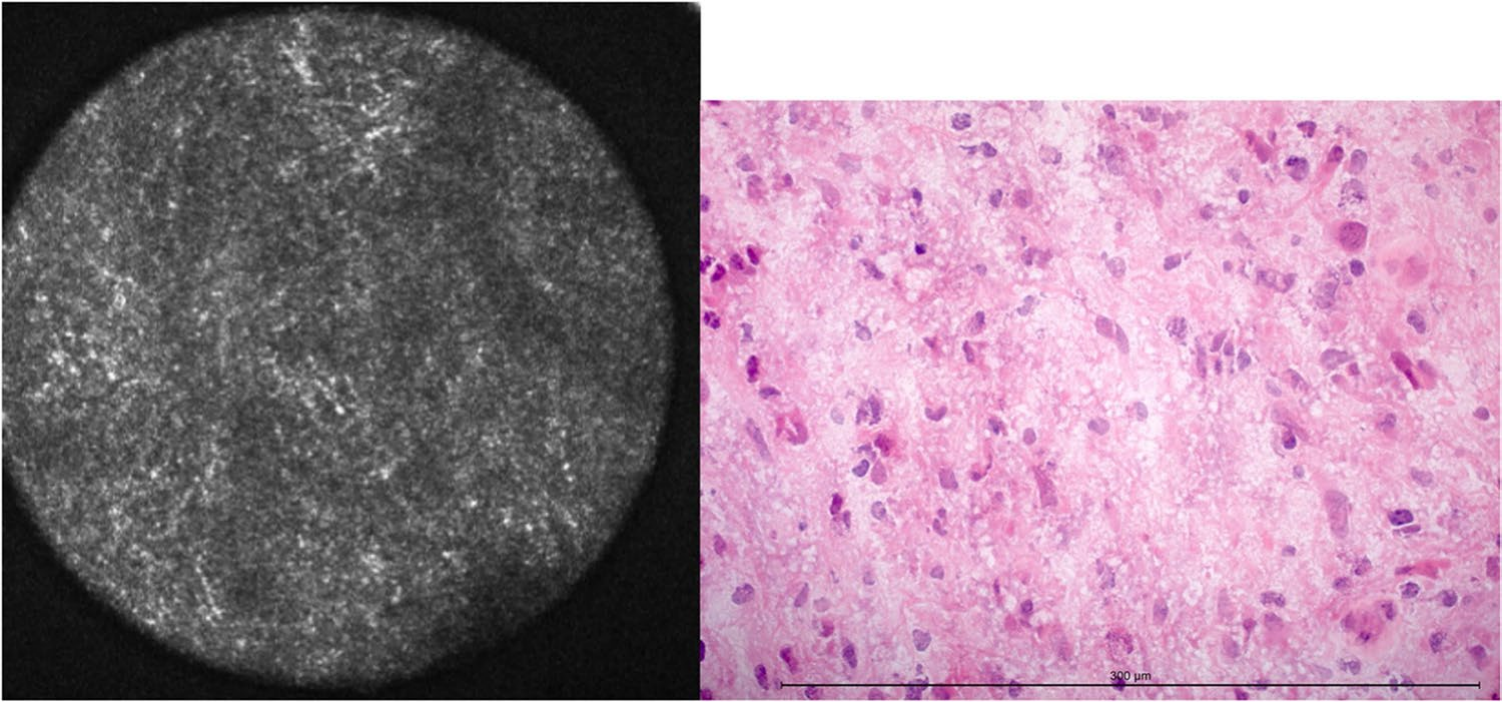
Glial matrix changes in CLE and H&E-frozen section. The diameter of the confocal image is 300 μm. The length of the measuring bar is 300 μm

Reduction or loss of the glial fibrillary pattern (Fig. 3) as described above were interpreted as changes in the glial extracellular matter due to tumor infiltration. This correlates to the shortening and loss of processes in neoplastic astrocytes and thus rarefaction and microvacuolization of the glial matrix. It appeared as a visible discontinuity of the glial fibrillary pattern or even its complete absence. While a disrupted fibrillary pattern can be used to identify glial tumors, the absence of any fibrillary structures might complicate differentiation from other intracranial neoplasms.

Of 125 samples only 122 could be evaluated for changes of the glial matrix due to insufficient matrix staining in 3 samples. Of these 122 specimens, 44 showed a relevant rarefaction of the glial matter, and 78 did not. With the EndoMAG1, 94 of these 122 were identified correctly. This results in an accuracy of 73.9 % (Table 3).

#### Hypercellularity

**Fig. 4:**
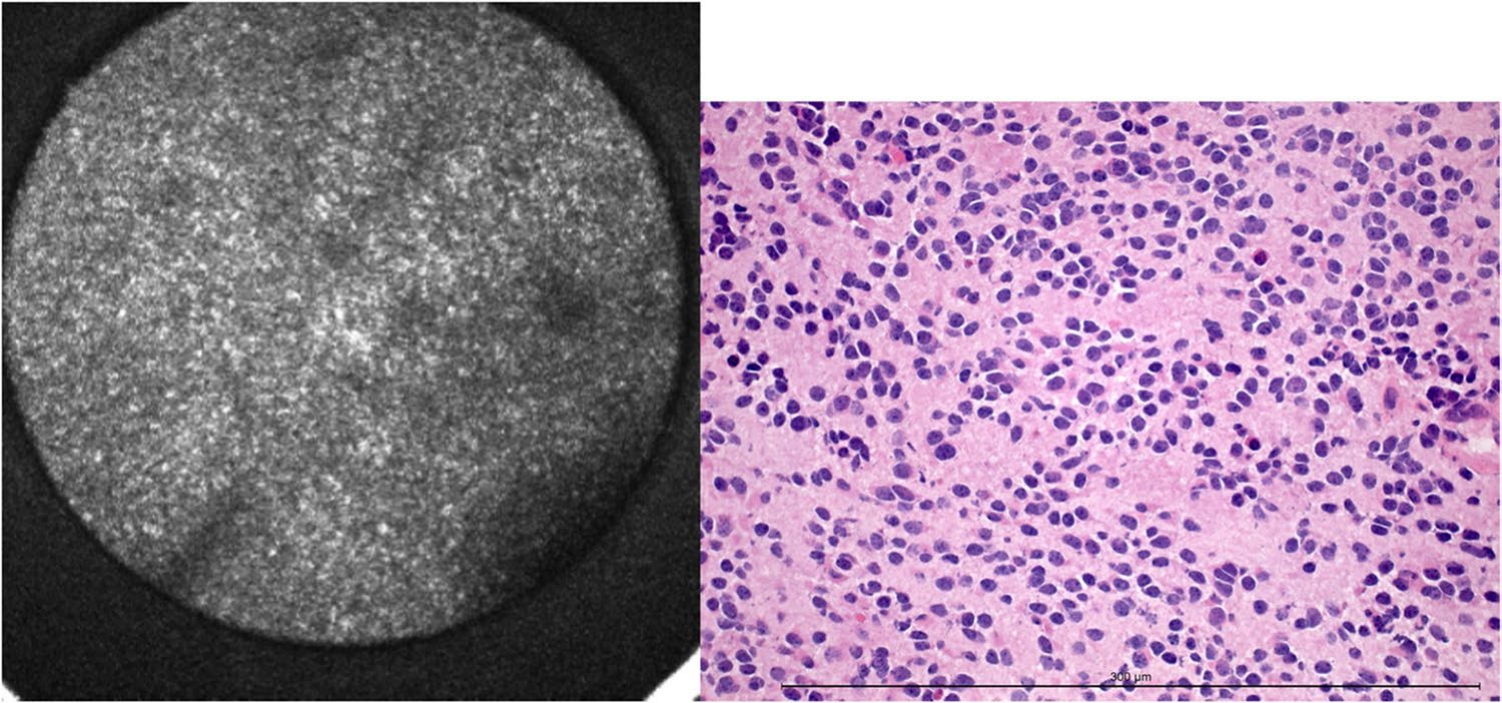
Hypercellularity in CLE and H&E-frozen section. The diameter of the confocal image is 300 μm. The length of the measuring bar is 300 μm

Hypercellularity presented as extensive, dense signal losses and disruptions of the fibrillary glial matrix (Fig. 4). These dark conglomerates were interpreted as groups of densely lying tumor cells. Single-cell margins could not be delineated in most cases with hypercellularity.

90 of 125 samples showed an increased cellularity, 35 did not. In total, 113 of these were attributed correctly via CLE, amounting to an accuracy of 90.4 % for hypercellularity (Table 3).

#### Nuclear pleomorphism

**Fig. 5:**
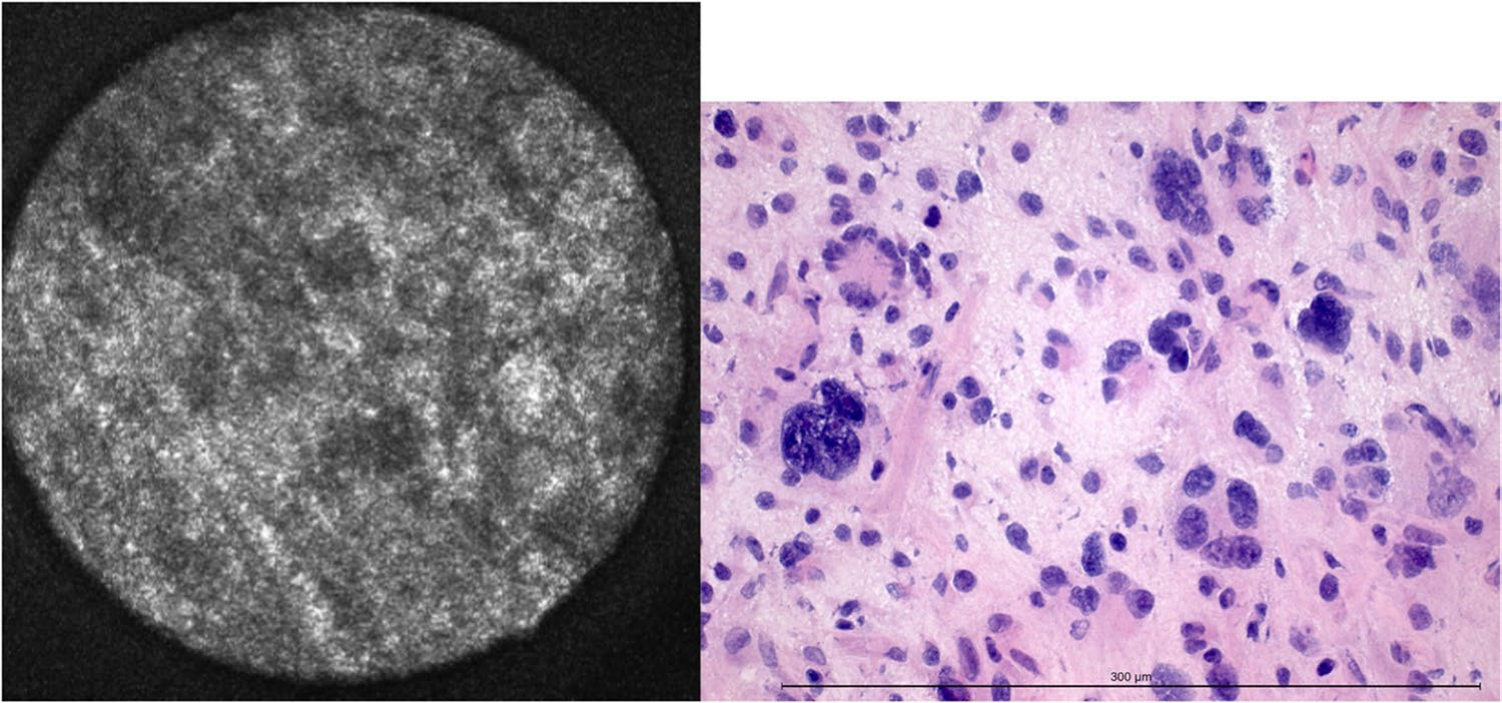
Nuclear pleomorphism in CLE and H&E-frozen section. The diameter of the confocal image is 300 μm. The length of the measuring bar is 300 μm

Variability in nuclear size and shape could be estimated by assessment of the irregularity or patchiness of the acquired CLE images (Fig. 5). Single nuclei or nucleoli could not be visualized by autofluorescence-based CLE and thus not be analyzed.

85 of 125 samples displayed pleomorphic nuclei, and 40 did not. With the confocal laser endomicroscope, 105 cases were identified correctly. Thus, the accuracy for nuclear pleomorphism was 84.0 % (Table 3).

#### Microvascular proliferation

**Fig. 6:**
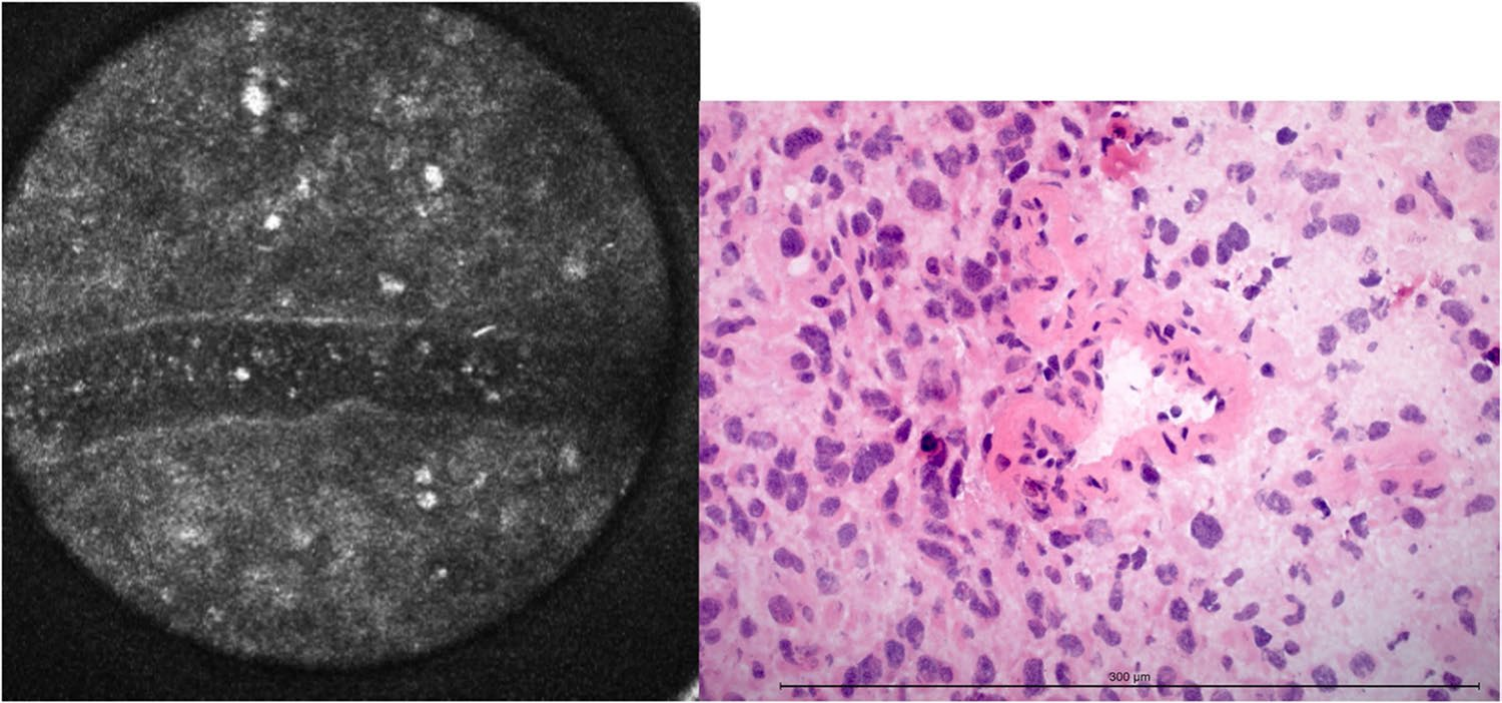
Hypervascularization in CLE and H&E-frozen section. The diameter of the confocal image is 300 μm. The length of the measuring bar is 300 μm

CLE showed enlarged blood vessels and vascular convolutes as irregularly traversing streaks. In some cases, the lumens of the vessels were visualized (Fig. 6) as containing very small, hyperintense structures interpreted as blood cells, though not specifically erythrocytes. Fine capillary networks as predominantly seen in oligodendrogliomas could also be identified. They presented as stretched, branchy structures with a diameter of about 10 μm, which fit the frozen section findings.

Sixty-one specimens showed microvascular proliferation, and 64 did not. In total, 99 of 125 were identified correctly via CLE, resulting in an accuracy of 79.2 % (Table 3).

#### Mitotic activity

**Fig. 7:**
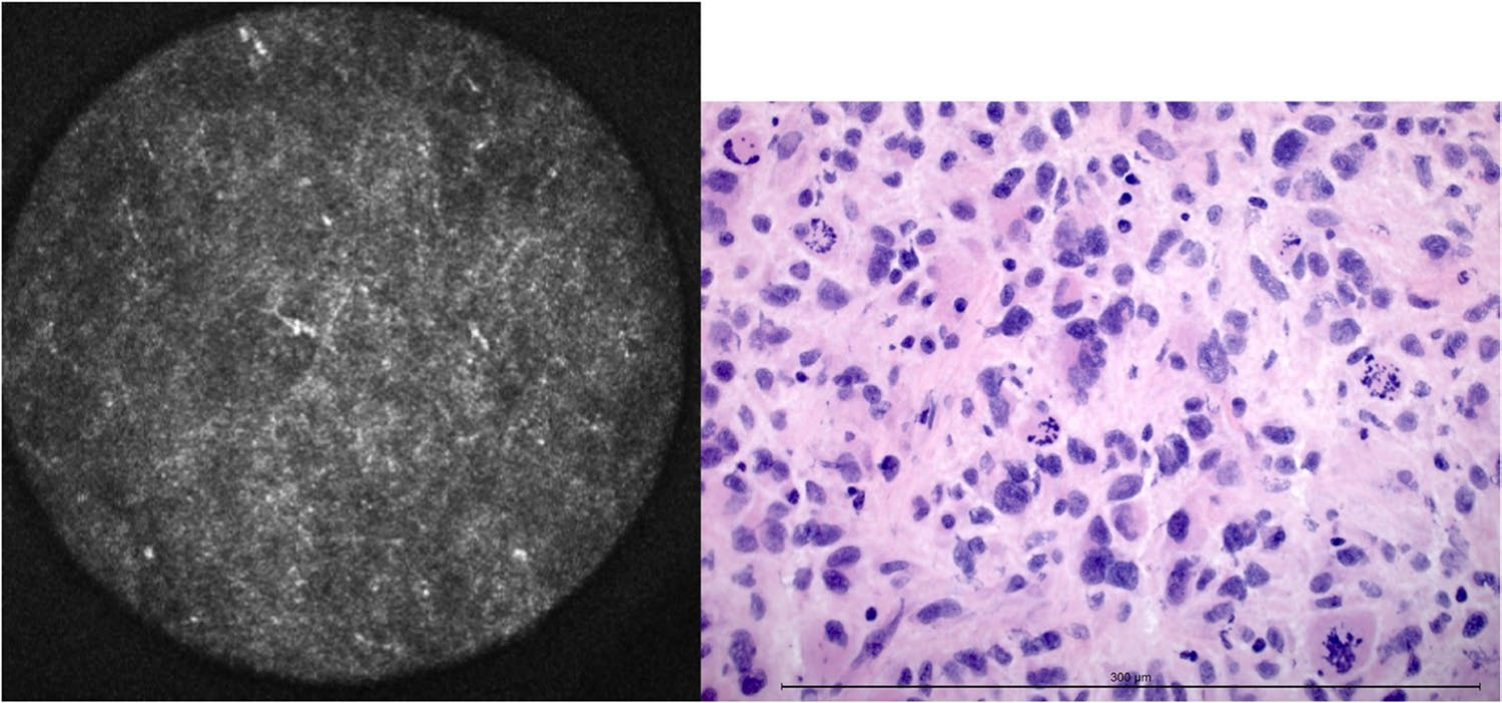
Mitotic figures in CLE and H&E-frozen section. The diameter of the confocal image is 300 μm. The length of the measuring bar is 300 μm

Although an important factor in determining malignancy in gliomas, we were not able to identify proliferating cells in mitosis via autofluorescence-based CLE even in samples with extreme mitotic activity (Fig. 7). Thus, no accuracy was calculated.

#### Necrosis

**Fig. 8:**
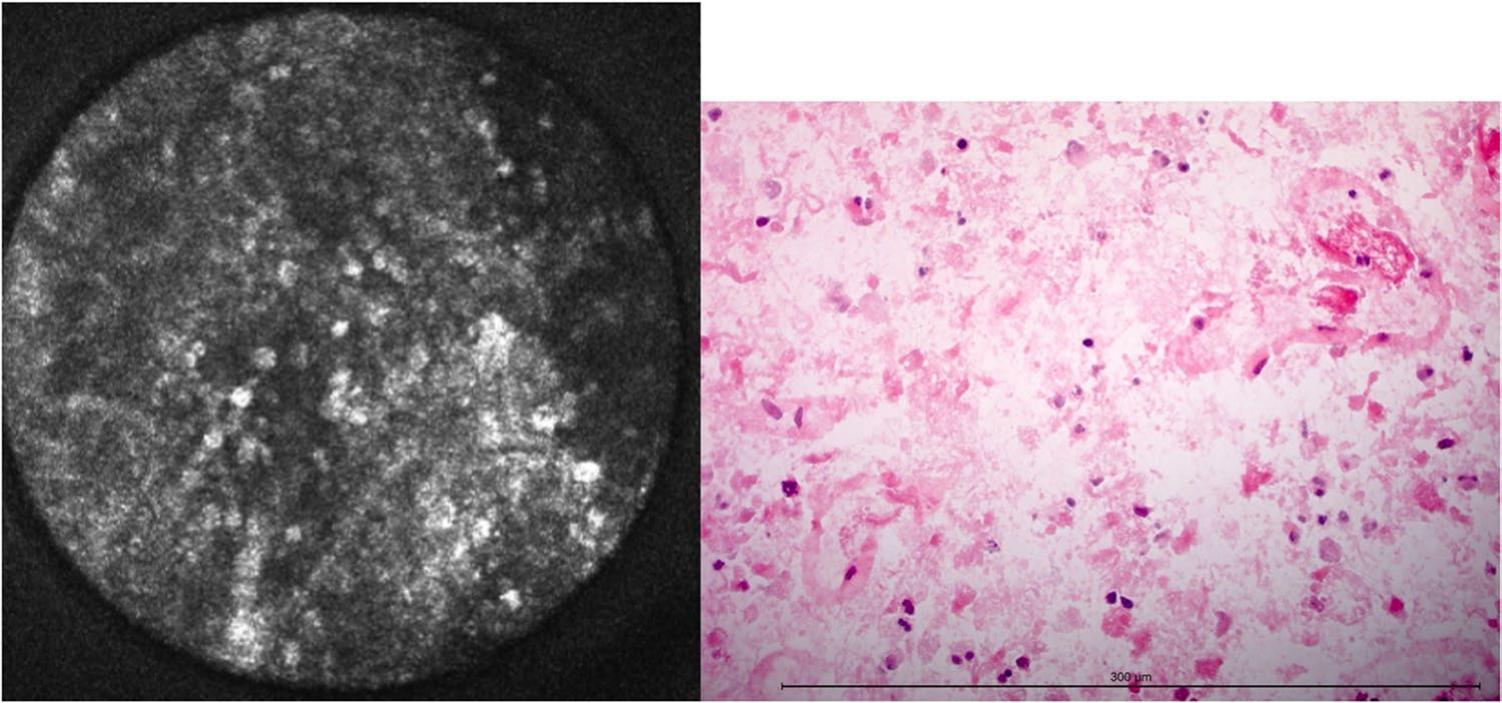
Necrosis in CLE and H&E-frozen section. The diameter of the confocal image is 300 μm. The length of the measuring bar is 300 μm

Necrosis appeared as a loss of fibrillary pattering with the simultaneous appearance of round, hyperintense structures with diameters between 3 and 20 μm (Fig. 8). Depending on the advance of disintegration of glial tumor matter, CLE showed only a few of these round formations or presented as large patchy areas that seemed only loosely interconnected. This went hand in hand with the disruption of the glial matrix, making it more difficult to state a cell of origin. Even though a pathognomonic histological finding for glioblastoma, pseudopalisading necrosis could not be differentiated from other types of necrosis via CLE.

Only 16 specimens contained necrotic tissue, 109 samples did not show necrosis. One hundred fifteen were matched correctly, the accuracy being 92.0 % (Table 3).

### Differentiation between tumor and surrounding tissue via CLE

One hundred three of 125 samples contained glial tumors. Twenty-two of the 125 samples only showed grey or white matter, partly with reactive changes. Via CLE, 102 of 125 samples were interpreted as tumorous. For tumors, CLE showed an accuracy of 91.2 %, a sensitivity of 94.17 %, a specificity of 77.27 %, a positive predictive value of 95.1 %, and a negative predictive value of 73.9 %.

**Fig. 9:**
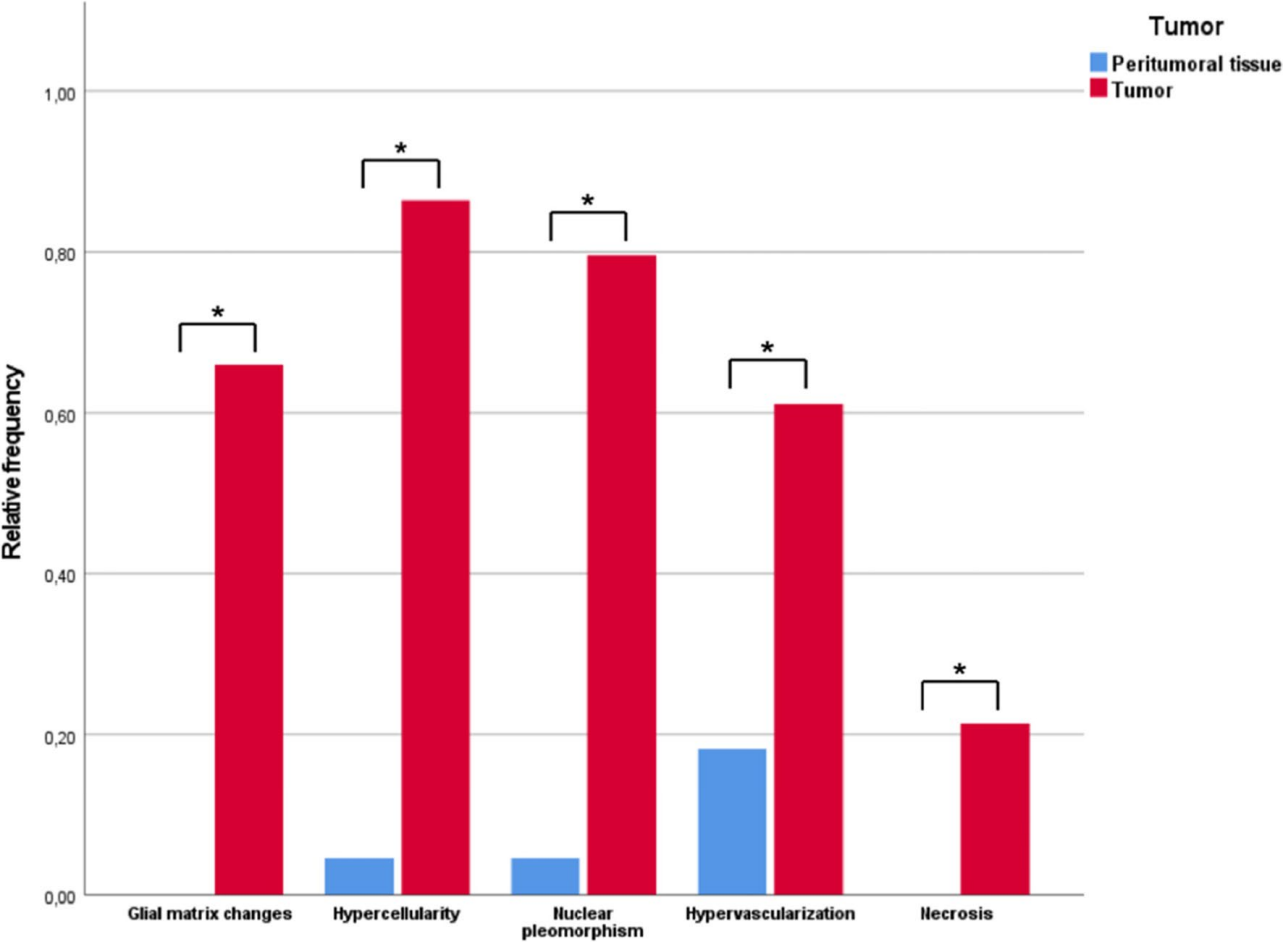
Relative frequencies of visualization of glial matrix changes, hypercellularity, nuclear pleomorphism, hypervascularization, and necrosis in tumor versus peritumoral tissue. Statistical significance was reached for each histomorphologic feature (*)

Via CLE, in 68 of 103 tumor samples changes of the glial matter were seen, and in zero of the non-tumorous samples (*p* < 0.001*, Fig. 9). Hypercellularity was seen in 82 of 103 tumor samples and 1 of 22 surrounding tissue samples via CLE (*p* < 0.001*). Nuclear pleomorphism was identified in 82 tumor samples and 1 non-tumor sample (*p* < 0.001*). Sixty-three tumor specimens and 4 grey/white matter samples showed hypervascularization in CLE (*p* < 0.001*). Via CLE, necrosis was identified in 22 tumor biopsies and zero surrounding brain tissue biopsies (*p* = 0.013*).

### Differentiation between astroglial and oligodendroglial tumors via CLE

#### Astroglial tumors

Of 125 samples, 102 were obtained from patients with astroglial tumors. Of these 102 specimens, 83 contained tumor tissue and 19 samples showed surrounding brain tissue. Accuracy for detection of tumor vs. non-tumor was 90.2 % for astroglial tumors. Sensitivity was 93.98 % and specificity was 73.68 %. The positive and negative predictive values were 93.98 % and 73.68 %, respectively.

#### Oligodendroglial tumors

The remaining 23 samples were obtained from patients with oligodendroglial tumors. Twenty of these 23 biopsies contained tumors, and 3 were tumor-free. Accuracy for differentiation between tumor and surrounding tissue via CLE was 95.65 %. Sensitivity was 100 %, specificity 95.0 %, the positive predictive value was 75.0 % and the negative predictive value for the tumor was 100 %.

#### Histological criteria to differentiate

Of all histological criteria analyzed beforehand (changes in the glial matrix, hypercellularity, nuclear pleomorphism, hypervascularization, and necrosis), the only statistically significant difference between astro- and oligodendroglial tumors specimens was a greater number of samples with higher nuclear pleomorphism in astrocytic gliomas than oligodendrogliomas (*p* < 0.001*). Astrocytic tumors also showed a higher number of specimens with changes in glial matter and necrosis than oligodendrogliomas, but this difference was not statistically significant. No difference was seen for hypercellularity and hypervascularization.

### Differentiating between low-grade and high-grade gliomas via CLE

#### Low-grade gliomas

Thirty-nine samples were obtained of patients with gliomas WHO grades 1–2, 32 of which contained tumor, and 7 were free of tumors. Accuracy for detection of tumor for low-grade gliomas via CLE was 88.1 %, sensitivity was 91.43 %, specificity was 71.43 %, and the positive and negative predictive values were 94.12 % and 62.5 %, respectively.

#### High-grade gliomas

Eighty-three specimens were collected from patients with gliomas WHO grades 3–4. Of these 83 specimens, 68 contained tumor tissue, 15 did not. Accuracy for differentiation between tumor and tumor-free samples via CLE was 92.77 % for high-grade gliomas. Sensitivity was 95.59 %, specificity was 80.0 %, the positive predictive value was 95.59 %, and the negative predictive values was 80.0 %.

#### Histological criteria to differentiate

Again, all histological criteria mentioned above were compared in visualization in low- and high-grade gliomas via CLE. No difference was shown for changes in glial extracellular matter. Hypercellularity and nuclear pleomorphism were greater in HGGs, hypervascularization was visualized more often in LGGs, each without statistical significance. Necrosis was shown to be the only statistically significant criterion to differentiate between low- and high-grade gliomas (*p* = 0.002*).

## Discussion

### Fluorescent dyes and their applicability with EndoMAG1

Various fluorescent dyes such as acridine orange, acriflavine, fluorescein sodium, cresyl violet, 5-aminolevulinic acid, sulforhodamine 101, and indocyanine green have been evaluated in rodent models for their practical use in visualizing glial brain tumors via CLE [9, 15]. Fluorescent dyes have to meet the following requirements: Ideally, the fluorescent dye accumulates selectively in tumor tissue, it has to be approved for human use, and its excitation and emission spectrum fits available confocal laser endomicroscopy. Of the aforementioned dyes, currently, only 5-ALA, FNa, and ICG are approved for neurosurgical use in humans in the EU and USA.

Regarding confocal laser endomicroscopy, fluorescent sodium is the most intensively studied fluorescent dye. Rodent models as well as human studies in ex vivo and in vivo settings have proven their ability to assist in the visualization of intracranial neoplasms. No clear benefit has been shown for CLE with 5-ALA yet, even though 5-ALA has the major advantage of being highly selective for malignant gliomas. Due to the excitation and emission spectra of both fluorescein sodium and 5-ALA [16, 17], these fluorescent dyes can only be used in combination with blue-laser confocal laser endomicroscopes.

Indocyanine green is most commonly used for intraoperative angiography in neurosurgical operating theaters. It is a relatively safe fluorescent dye with adverse drug reaction rates of less than 0.2 % [18]. Martirosyan et al. have shown that ICG can help visualize tumor margins via CLE in mice while acknowledging the disadvantage of ICG also accumulating in healthy brain tissue due to surgical trauma [15]. Another potential setback of ICG applicability is its accumulation behavior depending on the dosage. In humans, individual doses of 0.5 mg/kg bodyweight are approved for diagnostic uses, while the total daily dose should not surpass 5 mg/kg bodyweight. Unfortunately, Hansen et al. showed that doses of 60–120 mg/kg body weight were necessary for rats to achieve lasting ICG enrichment in glial tumors [19]. Another study by Haglund et al. demonstrated an ICG clearance within five to ten minutes at a dose of 1–2 mg/kg bodyweight [20]. These studies demonstrate the difficulty of dosing intravenous ICG when analyzing brain tissue using CLE. Further research should be done on how to dose and time its administration for CLE in brain tumor patients in an in vivo setting.

### Ex vivo analysis

Examining tissue specimens ex vivo is an acceptable compromise when analyzing tissue microstructure via CLE. Disadvantages of ex vivo analysis are a potentially greater surgical trauma to the brain tissue and resection of tissue that might be labeled as healthy afterward. In an experimental setting, when histological sections have to be prepared as a gold standard, this is an inevitable detriment. Another disadvantage of ex vivo CLE is the impossibility of visualizing vital brain tissue including blood flow. Belykh et al. have shown CLE to be of use in demonstrating normal blood flow in the CNS as well as pathologies such as thromboses and alterations in flow velocity [21]. This could be of great interest to visualize blood vessel pathologies in malignant gliomas.

Ex vivo analysis still offers benefits in the experimental setting. It allows tissue examination without prolonging surgery time and rapid investigation of several patients without the need for sterilization in between. The examiner is not bound to one surgical theater and thus, it is not necessary for the surgeon himself to handle CLE image acquisition and interpretation. At the moment, only very few surgeons are experienced in interpreting CLE images and thus CLE applicability in the operating room is limited.

Another advantage of ex vivo imaging is the guaranteed match between CLE imaging and histological sections. With in vivo imaging, there is a higher chance of acquiring CLE images in a slightly different spot than is later provided for histological processing. In the present study, the examiner paid attention to investigating the specimen completely. Still, both in vivo and ex vivo settings cannot capture every plane and a clear allocation of histological features to CLE images remains challenging.

CLE ex vivo imaging reduces motion artifacts, which have been described as a disadvantage for handheld confocal laser endomicroscopes [22, 23]. Martirosyan et al. describe the need for a higher number of CLE images when analyzing in vivo versus ex vivo, likely due to blood and motion artifacts [14]. In the present study, motion artifacts did not pose any challenge as the CLE was mounted to a fixed metal stand. Blood was cleared off the specimens using isotonic saline solution directly prior to analysis.

In conclusion, both ex vivo and in vivo examinations offer advantages in CLE imaging. In an experimental scope, ex vivo imaging is a reliable and practical approach to studying tissue microstructure in glial brain tumors. The long-term goal of CLE imaging remains in vivo data acquisition and interpretation.

### Differentiating between brain tissue and glial tumors by analyzing histological features

The development of reliable criteria is the prerequisite for standardized interpreting confocal images independent of the examiner.

The appearance of glial tissue in CLE imaging has been described as fibrillary prior to this study [11, 13, 24]. This is similar to our findings regarding the glial matrix, with an overall organized structure consistent with the microanatomy of grey and white matter. Differences in the presentation of grey versus white matter or healthy versus tumor tissue have not been stated in the aforementioned studies. Contrary to that, we have seen significant differences (*p* < 0.001) in glial structure in healthy brain tissue and glial tumors, mostly presenting as a loss or disruption of the described fibrillary structures. This was even more pronounced in high-grade than low-grade gliomas and in astroglial versus oligodendroglial tumors, without reaching statistical significance.

High cellularity and nuclear pleomorphism have been described as important diagnostic features in previous studies [8, 13–15, 24, 25]. Glial tumor has been characterized as an accumulation of irregularly shaped cells [11]. This description mostly matches our results, with tumor cells appearing as either round or irregularly shaped signal losses. In tumors with high cellularity, these cells formed visual agglomerates and cell borders could often not be delineated. Extensive cellularity or nuclear pleomorphism also resulted in a disruption of the fibrillary appearance of the glial tissue, a possible bias that has to be addressed in further studies. Both high cellularity and nuclear pleomorphism were shown to be reliable criteria in differentiating glial tumors and healthy brain tissue (*p* < 0.001 for each). This is concordant with findings by Belykh et al., who state that high cellularity was found significantly more often in glial tumor biopsies than in healthy or reactive brain tissue [10].

In the present study, low-grade tumors and high-grade tumors did not show a statistically significant difference in either cellularity or nuclear pleomorphism, whereas nuclear pleomorphism was significantly greater in astroglial versus oligodendroglial tumors (*p* < 0.001). Blinded studies are needed to evaluate whether nuclear pleomorphism can be used as a tool to differentiate between astrocytic and oligodendrocytic tumors.

Many studies have stated the visualization of necrotic tissue via CLE [13, 24], often lacking further description. Eschbacher et al. just state necrosis to be an acellular region [8], and Acerbi et al. have described it as amorphic tissue with low cellularity [25]. This only corresponded roughly to our findings. Interestingly enough, throughout the present study, necrosis presented as an accumulation of round formations with high signal intensity, most likely a representation of intracellular changes in cell death.

With the application of these histological features, CLE reached an accuracy of 91.2 %, a sensitivity of 94.17 %, and a specificity of 77.27 % for the identification of tumors. Acerbi et al. examined the accuracy of differentiation using FNa between tumor, normal or reactively altered tissue, infiltration zone, and necrosis in 15 patients with glioblastoma. The accuracy of tumor detection using CLE versus cryosection was only 60 % [25], suggesting that a separate assessment of histological features as visualized by CLE might facilitate differentiation between tumor and brain tissue. Yet, the gold standard of tissue visualization remains the microscopic evaluation of paraffin sections. Asano et al. examined edges of glial tumors: The sensitivity of the frozen section examination compared to the paraffin sections for tumor versus no tumor was 96.1 %, and the specificity 80.4 % [26].

Perioperatively, frozen sections are used to visualize tumor tissue by a neuropathologist. While processing times of 20–40 min are reported for frozen sections [27, 28], Acerbi et al. as well as Martirosyan et al. report biopsy evaluation times of less than 6 min with CLE [14, 25].

Finally, the economic aspect of incorporating a novel technique is of high relevance to surgical departments. With CLE becoming a more and more competitive technique and more manufacturers taking an interest in CLE for neurosurgery, a further increase in imaging quality and the emergence of more cost-effective models is to be expected. At the moment, high procurement costs of CLE setups might prevent hospitals, mostly in countries of the global south, from incorporating this new technique into their daily life.

### Study limitations

Many previous studies used different fluorescent agents and different confocal laser endomicroscopes with lasers of another color. This reduces the comparability of findings between the present study and previous studies, but also all studies in general. The CLE used in the present study utilizes a laser in the red spectrum, thus not compatible with fluorescein sodium, which is vastly used. It is also not approved for intraoperative use, thus limiting it to ex vivo studies.

This study was set up to collect extensive data on the appearance of glial brain tumors with respect to their heterogeneity in order to create a detailed and comprehensive basis for future projects. All findings of the present need to be further evaluated in a blinded fashion to confirm the reliability of the developed criteria. Another aim of CLE studies must be to broaden the spectrum of examiners to even out differences in experience, possibly assess learning curves, and confirm objectivity.

## Conclusion

The analysis of visualization of relevant histological criteria of glial tumors is the groundwork for differentiating between healthy brain tissue and glial tumors via CLE, thus providing a safe way to harvest tissue for histopathological and genetical analysis of the tumor, and possibly maximizing the extent of resection in neurooncologic surgery. In this study, red laser CLE without a fluorescent agent did not provide inferior results to studies that used fluorescent agents.

## Data Availability

Data were generated at the Departments of Neurosurgery and Neuropathology of Saarland University Medical Center. Digital data supporting the findings of this study are available from the corresponding author [JO] on request. Non-digital data and materials supporting this study are stored at the Departments of Neurosurgery and Neuropathology of Saarland University Medical Center.

## References

[1] Sanai N, Polley M-Y, McDermott MW, Parsa AT, Berger MS (2011) An extent of resection threshold for newly diagnosed glioblastomas. J Neurosurg 115:3–8. 10.3171/2011.2.jns10998 21417701 10.3171/2011.2.jns10998

[2] Chaichana KL, Cabrera-Aldana EE, Jusue-Torres I, Wijesekera O, Olivi A, Rahman M, Quinones-Hinojosa A (2014) When gross total resection of a glioblastoma is possible, how much resection should be achieved? World Neurosurg 82:e257–e265. 10.1016/j.wneu.2014.01.019 24508595 10.1016/j.wneu.2014.01.019

[3] Albuquerque LAF, Almeida JP, de Macêdo Filho LJM, Joaquim AF, Duffau H (2021) Extent of resection in diffuse low-grade gliomas and the role of tumor molecular signature—a systematic review of the literature. Neurosurg Rev 44:1371–1389. 10.1007/s10143-020-01362-8 32770298 10.1007/s10143-020-01362-8

[4] Smith JS, Chang EF, Lamborn KR, Chang SM, Prados MD, Cha S, Tihan T, Vandenberg S, McDermott MW, Berger MS (2008) Role of extent of resection in the long-term outcome of low-grade hemispheric gliomas. J Clin Oncol 26:1338–1345. 10.1200/JCO.2007.13.9337 18323558 10.1200/JCO.2007.13.9337

[5] Ellingson BM, Abrey LE, Nelson SJ, Kaufmann TJ, Garcia J, Chinot O, Saran F, Nishikawa R, Henriksson R, Mason WP, Wick W, Butowski N, Ligon KL, Gerstner ER, Colman H, de Groot J, Chang S, Mellinghoff I, Young RJ et al (2018) Validation of postoperative residual contrast-enhancing tumor volume as an independent prognostic factor for overall survival in newly diagnosed glioblastoma. Neuro-Oncology 20:1240–1250. 10.1093/neuonc/noy053 29660006 10.1093/neuonc/noy053 PMC6071654

[6] Grabowski MM, Recinos PF, Nowacki AS, Schroeder JL, Angelov L, Barnett GH, Vogelbaum MA (2014) Residual tumor volume versus extent of resection: predictors of survival after surgery for glioblastoma. J Neurosurg 121:1115–1123. 10.3171/2014.7.JNS132449 25192475 10.3171/2014.7.JNS132449

[7] Breuskin D, DiVincenzo J, Kim Y-J, Urbschat S, Oertel J (2013) Confocal laser endomicroscopy in neurosurgery: a new technique with much potential. Minim Invasive Surg 2013:1–5. 10.1155/2013/851819 10.1155/2013/851819 PMC3745972 23984062

[8] Eschbacher J, Martirosyan NL, Nakaji P, Sanai N, Preul MC, Smith KA, Coons SW, Spetzler RF (2012) In vivo intraoperative confocal microscopy for real-time histopathological imaging of brain tumors: Clinical article. JNS 116:854–860. 10.3171/2011.12.JNS11696 10.3171/2011.12.JNS11696 22283191

[9] Martirosyan NL, Georges J, Eschbacher JM, Cavalcanti DD, Elhadi AM, Abdelwahab MG, Scheck AC, Nakaji P, Spetzler RF, Preul MC (2014) Potential application of a handheld confocal endomicroscope imaging system using a variety of fluorophores in experimental gliomas and normal brain. Neurosurg Focus 36:E16. 10.3171/2013.11.FOCUS13486 24484254 10.3171/2013.11.FOCUS13486

[10] Belykh E, Zhao X, Ngo B, Farhadi DS, Byvaltsev VA, Eschbacher JM, Nakaji P, Preul MC (2020) Intraoperative confocal laser endomicroscopy ex vivo examination of tissue microstructure during fluorescence-guided brain tumor surgery. Front Oncol 10:599250. 10.3389/fonc.2020.599250 33344251 10.3389/fonc.2020.599250 PMC7746822

[11] Foersch S, Heimann A, Ayyad A, Spoden GA, Florin L, Mpoukouvalas K, Kiesslich R, Kempski O, Goetz M, Charalampaki P (2012) Confocal laser endomicroscopy for diagnosis and histomorphologic imaging of brain tumors in vivo. PLoS One 7:e41760. 10.1371/journal.pone.0041760 22911853 10.1371/journal.pone.0041760 PMC3404071

[12] Breuskin D, Szczygielski J, Urbschat S, Kim Y-J, Oertel J (2017) Confocal laser endomicroscopy in neurosurgery-an alternative to instantaneous sections? World Neurosurg 100:180–185. 10.1016/j.wneu.2016.12.128 28069420 10.1016/j.wneu.2016.12.128

[13] Charalampaki P, Javed M, Daali S, Heiroth H-J, Igressa A, Weber F (2015) Confocal laser endomicroscopy for real-time histomorphological diagnosis: our clinical experience with 150 brain and spinal tumor cases. Neurosurgery 62:171–176. 10.1227/NEU.0000000000000805 26181939 10.1227/NEU.0000000000000805

[14] Martirosyan NL, Eschbacher JM, Kalani MYS, Turner JD, Belykh E, Spetzler RF, Nakaji P, Preul MC (2016) Prospective evaluation of the utility of intraoperative confocal laser endomicroscopy in patients with brain neoplasms using fluorescein sodium: experience with 74 cases. Neurosurg Focus 40:E11. 10.3171/2016.1.FOCUS15559 26926051 10.3171/2016.1.FOCUS15559

[15] Martirosyan NL, Cavalcanti DD, Eschbacher JM, Delaney PM, Scheck AC, Abdelwahab MG, Nakaji P, Spetzler RF, Preul MC (2011) Use of in vivo near-infrared laser confocal endomicroscopy with indocyanine green to detect the boundary of infiltrative tumor: Laboratory investigation. J Neurosurg 115:1131–1138. 10.3171/2011.8.JNS11559 21923240 10.3171/2011.8.JNS11559

[16] Zehri A, Ramey W, Georges J, Mooney M, Martirosyan N, Preul M, Nakaji P (2014) Neurosurgical confocal endomicroscopy: a review of contrast agents, confocal systems, and future imaging modalities. Surg Neurol Int 5:60. 10.4103/2152-7806.131638 24872922 10.4103/2152-7806.131638 PMC4033764

[17] Zhang N, Tian H, Huang D, Meng X, Guo W, Wang C, Yin X, Zhang H, Jiang B, He Z, Wang Z (2017) Sodium fluorescein-guided resection under the YELLOW 560 nm surgical microscope filter in malignant gliomas: our first 38 cases experience. Biomed Res Int 2017:7865747. 10.1155/2017/7865747 29124069 10.1155/2017/7865747 PMC5662847

[18] Hope-Ross M, Yannuzzi LA, Gragoudas ES, Guyer DR, Slakter JS, Sorenson JA, Krupsky S, Orlock DA, Puliafito CA (1994) Adverse reactions due to indocyanine green. Ophthalmology 101:529–533. 10.1016/s0161-6420(94)31303-0 8127574 10.1016/s0161-6420(94)31303-0

[19] Hansen DA, Spence AM, Carski T, Berger MS (1993) Indocyanine green (ICG) staining and demarcation of tumor margins in a rat glioma model. Surg Neurol 40:451–456. 10.1016/0090-3019(93)90046-4 7694381 10.1016/0090-3019(93)90046-4

[20] Haglund MM, Berger MS, Hochman DW (1996) Enhanced optical imaging of human gliomas and tumor margins. Neurosurgery 38:308–317. 10.1097/00006123-199602000-00015 8869058 10.1097/00006123-199602000-00015

[21] Belykh E, Zhao X, Ngo B, Farhadi DS, Kindelin A, Ahmad S, Martirosyan NL, Lawton MT, Preul MC (2021) Visualization of brain microvasculature and blood flow in vivo: Feasibility study using confocal laser endomicroscopy. Microcirculation 28. 10.1111/micc.12678 10.1111/micc.12678 33426724

[22] Aubreville M, Stoeve M, Oetter N, Goncalves M, Knipfer C, Neumann H, Bohr C, Stelzle F, Maier A (2019) Deep learning-based detection of motion artifacts in probe-based confocal laser endomicroscopy images. Int J Comput Assist Radiol Surg 14:31–42. 10.1007/s11548-018-1836-1 30078151 10.1007/s11548-018-1836-1

[23] Dittberner A, Ziadat R, Hoffmann F, Pertzborn D, Gassler N, Guntinas-Lichius O (2021) Fluorescein-guided panendoscopy for head and neck cancer using handheld probe-based confocal laser endomicroscopy: a pilot study. Front Oncol 11:671880. 10.3389/fonc.2021.671880 34195078 10.3389/fonc.2021.671880 PMC8236705

[24] Forest F, Cinotti E, Yvorel V, Habougit C, Vassal F, Nuti C, Perrot J-L, Labeille B, Péoc’h M (2015) Ex vivo confocal microscopy imaging to identify tumor tissue on freshly removed brain sample. J Neuro-Oncol 124:157–164. 10.1007/s11060-015-1832-z 10.1007/s11060-015-1832-z 26033548

[25] Acerbi F, Pollo B, De Laurentis C, Restelli F, Falco J, Vetrano IG, Broggi M, Schiariti M, Tramacere I, Ferroli P, DiMeco F (2020) Ex vivo fluorescein-assisted confocal laser endomicroscopy (CONVIVO® System) in patients with glioblastoma: results from a prospective study. Front Oncol 10:606574. 10.3389/fonc.2020.606574 33425764 10.3389/fonc.2020.606574 PMC7787149

[26] Asano K, Kurose A, Kamataki A, Kato N, Ogawa K, Katayama K, Kakuta K, Fumoto T, Ohkuma H (2018) Importance and accuracy of intraoperative frozen section diagnosis of the resection margin for effective carmustine wafer implantation. Brain Tumor Pathol 35:131–140. 10.1007/s10014-018-0320-5 29948295 10.1007/s10014-018-0320-5

[27] Chen D, Nauen DW, Park H-C, Li D, Yuan W, Li A, Guan H, Kut C, Chaichana KL, Bettegowda C, Quiñones-Hinojosa A, Li X (2021) Label-free imaging of human brain tissue at subcellular resolution for potential rapid intra-operative assessment of glioma surgery. Theranostics 11:7222–7234. 10.7150/thno.59244 34158846 10.7150/thno.59244 PMC8210590

[28] Eichberg DG, Shah AH, Di L, Semonche AM, Jimsheleishvili G, Luther EM, Sarkiss CA, Levi AD, Gultekin SH, Komotar RJ, Ivan ME (2021) Stimulated Raman histology for rapid and accurate intraoperative diagnosis of CNS tumors: prospective blinded study. J Neurosurg 134:137–143. 10.3171/2019.9.JNS192075 31812144 10.3171/2019.9.JNS192075

